# Underutilized *Crocus Sativus* L. Flowers: A Hidden Source of Sustainable High Value-Added Ingredients

**DOI:** 10.1007/s11130-023-01065-7

**Published:** 2023-06-30

**Authors:** Débora Cerdá-Bernad, Estefanía Valero-Cases, Francisca Pérez-Llamas, Joaquín Julián Pastor, María José Frutos

**Affiliations:** 1 Agro-Food Technology Department, CIAGRO-UMH, Miguel Hernández University, Orihuela, 03312 Spain; 2 Physiology Department, University of Murcia, Murcia, 30100 Spain; 3 Engineering Department, CIAGRO-UMH, Miguel Hernández University, Orihuela, 03312 Spain

**Keywords:** Saffron flower, Bio-residues, Food composition, Sustainability, Functional ingredients

## Abstract

*Crocus sativus* L. is used as a spice due to its organoleptic characteristics. Only flower stigmas are used for its production, as the rest of the flower is discarded as waste. This fact represents a lack of sustainability, since around 230,000 flowers are necessary to produce 1 kg of saffron. The main aim of this study was to contribute to the valorization of *Crocus sativus* L. spice and its floral by-products, through the study of their nutritional value and composition, in terms of hydrophilic and lipophilic compounds, as well as their functional properties. The results showed that saffron stigmas and floral bio-residues presented high contents of fiber, and the most abundant macronutrient were the carbohydrates, followed by proteins, and a low content in fats. All samples had high concentrations of glucose, fructose, lactic and malic acids, and minerals, mainly K, Ca and Mg. Furthermore, the polyunsaturated fatty acids were predominant, being linoleic acid (C18:2n6) the most abundant. Therefore, this research provides more in-depth information about the composition of saffron stigmas and floral by-products, to be considered as promising sources for the development of functional ingredients with new applications in the food industry.

**Supplementary Information:**

The online version contains supplementary material available at 10.1007/s11130-023-01065-7.

## Introduction

Saffron (*Crocus sativus* L.), a monocotyledonous herbaceous plant from the Iridaceae family, produces the most expensive spice in the world. The high costs are because producing 1 kg of saffron is necessary around 230,000 flowers (⁓60–80 kg, being ⁓78% tepals), as only the dried-up stigmas are used for the spice [1]. The rest of the flower is discarded. Therefore, considering that hundreds of kilograms (⁓205,000 kg) of saffron arrive to the market each year and the production yields range between 0.02 and 0.03 kg of dry stigmas *per* hectare, the current production system is generating several hundreds of tons of tepal wastes (⁓9,500 − 12,700 tons/year) [2].

The chemical characteristics of saffron depend on the different geoclimatic conditions and processing techniques used by the growers. However, this plant, originated from southern Europe and south-west Asia, is widely distributed in different areas, due to its high adaptability for cultivation [3]. The chemical composition of saffron is complex, having primary macromolecules, mostly carbohydrates, amino acids and proteins, and lower contents of minerals, fats and vitamins; and secondary metabolites such as carotenoids, monoterpenes, and flavonoids [4, 5]. These bioactive compounds are natural antioxidants that could prevent certain diseases related to the oxidative stress, such as cancer, metabolic syndrome, neurological disorders, among others [6]. However, the demonstration of tepals specific activity would convert this waste product into a high value ingredient, increasing the resource efficiency and the competitiveness of this traditional sector. Furthermore, their possible nutritional value may contribute to reducing malnutrition and hunger in the world, using them as a new source of food. Currently, hunger and undernourishment are on the rise, affecting many millions of people around the world, especially in developing countries, so there is a need to explore novel sustainable sources that will decrease this prevalence and have a positive social impact [7]. As a consequence, the main aim of this research was to contribute to the valorization of *Crocus sativus* L. and its floral by-products through their nutritional characterization to provide more in-depth information to develop innovative food ingredients with novel applications that could potentially increase saffron demand on the market and have economic, social and environmental benefits. The proximal and dietary fiber composition, minerals, organic acids, sugar contents, and the lipidic profile were determined in saffron stigmas and saffron floral by-products. Besides, the functional properties of saffron floral by-products were also evaluated. As far as we know, no research has been published to date that delves into the content of lipophilic compounds like the fatty acid profile and the functional properties of saffron floral biowaste.

## Materials and Methods

The section of material and methods is provided as Supplementary Materials.

## Results and Discussion

### Proximate Composition and Dietary Fiber Content of Saffron and its Floral By-Products

Data on the nutritional composition, energetic value and fiber composition of saffron floral by-products and saffron stigmas of different origins are presented in Table 1. Regarding the moisture, an important indicator of shelf life for food, the lyophilized flowers presented lower moisture than saffron stigmas, since the initial moisture values of SFL1 and SFL2 samples before drying were higher than 80 g *per* 100 g of fw (85.45 ± 0.51 for SFL1 and 86.81 ± 0.30 for SFL2). Saffron stigmas had higher moisture contents because they were dehydrated by air under low temperatures (< 60 °C). Spanish, Iranian and Greek saffron showed values lower than 12 g/100 g on dry weight, according to the maximum moisture allowed for saffron threads [8]. Thus, the moisture of all vegetal samples could be adequate for the storage to avoid deterioration because of microbiological, or chemical spoilage.

With respect to ash contents, which provide a measure of the total amount of minerals within a food, SFL2 and saffron stigmas samples presented ash values lower than 8 g/100 g on dry weight, according to the maximum total ash allowed for saffron spice, except for SFL1 (8.39 ± 0.00 g/100 g dw) showing statistically significant differences with the rest the samples (values around 5 g/100 g dw) [8].

**Table 1:** Proximal composition and dietary fiber in saffron and its floral by-products

	Samples				
	SFL1	SFL2	Spanish Saffron	Iranian Saffron	Greek Saffron
**Nutritional value (g/100 g dw)**					
Moisture	6.42 ± 0.68bc	5.52 ± 1.63c	8.90 ? 0.14ab	9.01 ? 0.05ab	10.74 ± 0.55a
Ash	8.39 ± 0.00a	4.89 ± 0.31b	5.59 ± 0.09b	5.57 ± 0.26b	4.81 ± 0.14b
Proteins	8.58 ± 0.08d	8.68 ± 0.02d	13.15 ± 0.13b	13.61 ± 0.13a	12.29 ± 0.02c
Lipids	5.81 ± 0.01ab	4.56 ± 0.77b	7.01 ± 0.29a	6.08 ± 0.17ab	6.22 ± 0.20a
Available carbohydrates	70.80 ± 0.75b	76.35 ± 2.07a	65.26 ± 0.25c	65.73 ± 0.16c	65.94 ± 0.23c
**Energy (kcal/100 g dw)**	369 ± 3b	381 ± 1a	377 ± 2ab	372 ± 2ab	369 ± 3b
**Fiber composition (g/100 g dw)**					
Total DF	26.59 ± 1.30a	22.56 ± 0.27ab	17.86 ± 2.64b	21.17 ± 0.02ab	17.64 ± 2.51b
Insoluble DF	19.07 ± 0.59a	20.24 ± 1.11a	14.61 ± 0.88b	14.55 ± 0.04b	15.15 ± 0.21b
Soluble DF	7.51 ± 0.71a	2.32 ± 1.38c	3.25 ± 1.76bc	6.61 ± 0.06ab	4.26 ± 0.21abc
**Inulin content (g/100 g dw)**	1.46 ± 0.47a	1.60 ± 0.51a	1.60 ± 0.65a	1.59 ± 0.15a	1.81 ± 0.67a

Means ± standard deviation in the same line followed by different lowercase letters indicate statistically significant differences at (*p* ≤ 0.05) for each sample (*n* = 3); SFL1, SFL2: Saffron floral by-products from two different producers

Available carbohydrates were the most abundant macronutrients in all the saffron stigmas and flower samples, followed by proteins and the lowest content was for lipids. Flowers, specially SFL2, had amounts of available carbohydrates significantly higher than saffron stigmas (76 g/100 g dw and 65 g/100 g dw, respectively) (Table 1). However, saffron stigmas showed significantly higher protein content (around 13 g/100 g dw), especially Iranian saffron, than flowers (around 8 g/100 g dw). Thus, saffron presented high amounts of protein which is a good source of amino acids for vital physiological functions. Regarding the energy values and lipids, any statistically significant differences were observed in the flowers (5 g/100 g dw of lipids) when compared to stigmas (around 6 g/100 g dw of lipids). These results were in accordance with the ones described by Serrano-Díaz et al. [9] who reported similar values of the proximal composition in saffron bio-residues and saffron stigmas from Castilla-La Mancha (Spain). Besides, these values were comparable with those obtained in the study of Muzaffar et al. [10] regarding Indian saffron stigmas. Furthermore, saffron flowers presented similar proximal composition compared to other edible flowers such as petals of different species (dahlia, rose, calendula and centaurea) that contained carbohydrates as the most abundant macronutrient (81–88%) followed by proteins (6–7%) and the lowest content in fat (0.1-5%) [11].

In addition, the dietary fiber was studied. The consumption of DF has positive effects on human digestive health. Total DF was higher in flowers (22–26 g/100 g dw) than in saffron stigmas (17–21 g/100 g dw). The insoluble DF was the major fraction of the total DF in all samples studied, being significantly higher in the floral by-products (20 g/100 g dw) than in saffron stigmas (15 g/100 g dw). These results were in accordance to those reported by Serrano-Díaz et al. [9] in which saffron and its bio-residues from Spain presented higher content of insoluble DF than soluble DF, being lower in stigmas. Regarding the values of soluble DF, SFL1 had a significant higher content (7 g/100 g dw) compared to SFL2 (2 g/100 g dw) and saffron stigmas from Iran showed a higher soluble DF content (6 g/100 g dw) respect to Spanish (3 g/100 g dw) and Greek saffron (4 g/100 g dw). The differences shown regarding the proximal composition and DF between SFL1 and SFL2 from Spain and between stigmas from Spain, Iran and Greece may be due to the growing conditions, such as temperature, humidity, soil properties, which could noticeably influence the chemical constituents of the plant [12]. Moreover, the content of inulin (as part of the soluble dietary fiber) was studied. Inulin, found naturally in many food plants, is usually used as dietary fiber and as a prebiotic in functional food, since it is not hydrolysed by human intestinal enzymes. Saffron and its floral by-products presented inulin content in similar amounts (1.50 g/100 g dw), showing statistically no-significant differences, suggesting that they could be used as a good source of prebiotic ingredients, stimulating the proliferation of the intestinal microbiota. These values were similar than that found in rye (0.5-1%), barley (0.5–1.5%) and wheat (1–4%) cereals [13].

### Mineral Composition of Saffron and its Floral By-Products

The mineral content of saffron and its floral by-products is shown in Table 2. Macrominerals, such as Ca, K, Na and Mg, are relevant for several physiological functions, both in plants and animals. The most abundant mineral in all saffron and its floral by-products samples was K, which may protect against bone loss and reduce the risk of cardiovascular diseases. The K content was significantly higher in saffron floral by-products (around 1500 mg/100 g dw) when compared to the saffron stigmas (around 1000 mg/100 g dw). These values represent 31.91% and 21.28% approximately of the daily intake of potassium in adults (4700 mg *per* day) with a consumption of 100 g of flowers or stigmas, respectively [14]. Besides, SFL1 presented higher amount of Ca (415.20 ± 25.46 mg/100 g dw) respect to SFL2 (112.60 ± 1.98 mg/100 g dw), showing statistically significant differences. Saffron stigmas had a low amount of Ca (around 86–120 mg/100 g dw). These values represent 31.94 and 9.23% approximately of the daily intake of calcium in adults (1300 mg per day) with a consumption of 100 g of flowers or stigmas, respectively [14]. Calcium also plays an important role in the human health especially in the prevention of bone loss and osteoporosis. Regarding Mg, SFL1 presented a significantly higher content (120.30 ± 4.95 mg/100 g dw) respect to SFL2 (103.30 ± 2.68 mg/100 g dw). Spanish and Iranian saffron stigmas showed similar values that SFL2. However, Greek saffron presented the lowest amount (88.70 ± 4.60 mg/100 g dw). These values represent around 28.64 and 24.40% of the daily intake of magnesium in adults (420 mg *per* day) with a consumption of 100 g of flowers or stigmas, respectively [14]. The values of Na were low in all samples, showing statistically no-significant differences between them, being around 9 mg/100 g dw in saffron flowers and 33 mg/100 g dw in stigmas from Iran and Greece, and 19 mg/100 g dw in stigmas from Spain. Fahim et al. [15] also reported high content of K (542.13 ± 0.01 mg/100 g) and Ca (486.25 ± 0.12 mg/100 g) in Iranian saffron petals and low amounts of Na (25.75 ± 0.01 mg/100 g). Regarding the microminerals, the concentration in all samples of Fe, an essential element for human physiology involved in several metabolic processes, was the highest, followed by Zn. SFL1 showed significantly higher levels of Fe (46.26 ± 1.44 mg/100 g dw) with respect to SFL2 (6.38 ± 0.03 mg/100 g dw). Iranian and Greek saffron stigmas presented similar values to SFL2, showing SFL2 statistically no-significant differences with stigmas. However, Spanish saffron presented the lowest concentration (5.45 ± 0.57 mg/100 g dw). These differences could be due to the different cultivation conditions, such as the mineral content in the soil, the use of some fertilizers or the relative bioaccumulation of this mineral in the plant. These values represent around 35.44 and 45.17% of the daily intake of iron in adults (18 mg per day) with a consumption of 100 g of flowers (SFL2) or stigmas, respectively [14]. With respect to Zn, SFL1 presented a significantly higher content (3.89 ± 0.27 mg/100 g dw) than SFL2 (2.10 ± 0.08 mg/100 g dw), which showed similar amounts to saffron stigmas. These values represent 35.36 and 24% approximately of the daily intake of zinc in adults (11 mg per day) with a consumption of 100 g of flowers or stigmas, respectively [14].

**Table 2:** Mineral composition in saffron and its floral by-products

	Samples				
	SFL1	SFL2	Spanish Saffron	Iranian Saffron	Greek Saffron
**Macrominerals (mg/100 g dw)**					
Ca	415.20 ± 25.46a	112.60 ± 1.98b	118.30 ± 25.10b	86.25 ± 6.01b	123.30 ± 63.98b
K	1530 ± 16a	1450 ± 51a	1114 ± 63bc	1136 ± 18b	971.30 ± 31.82c
Mg	120.30 ± 4.95a	103.30 ± 2.68b	100.20 ± 1.77bc	102.50 ± 1.41b	88.70 ± 4.60c
Na	9.00 ± 3.53a	9.20 ± 1.55a	19.00 ± 1.25a	33.75 ± 2.89a	33.25 ± 3.45a
**Microminerals (mg/100 g dw)**					
Cu	0.57 ± 0.01b	0.55 ± 0.04b	0.35 ± 0.07bc	0.25 ± 0.00c	1.63 ± 0.11a
Fe	46.26 ± 1.44a	6.38 ± 0.03bc	5.45 ± 0.57c	12.28 ± 3.29b	8.13 ± 1.03bc
Mn	2.51 ± 0.21a	0.95 ± 0.01b	1.15 ± 0.01b	1.30 ± 0.00b	1.25 ± 0.07b
Zn	3.89 ± 0.27a	2.10 ± 0.08b	2.65 ± 0.35ab	2.13 ± 0.53b	1.98 ± 0.46b

Means ± standard deviation in the same line followed by different lowercase letters indicate statistically significant differences at (*p* ≤ 0.05) for each sample (n = 3); SFL1, SFL2: Saffron floral by-products from two different producers

Serrano-Díaz et al. [9] reported similar values of Fe in saffron stigmas from Spain (0.011 ± 0.001 g/100 g dw). The Mn and Cu contents were low in all samples, except for SFL1 that showed significantly higher concentrations of Mn (2.51 ± 0.21 mg/100 g dw) compared to SFL2 (0.95 ± 0.01 mg/100 g dw) and stigmas (1.15–1.30 mg/100 g dw). These values represent around 41.30 and 56.52% of the daily intake of manganese in adults (2.3 mg) with a consumption of 100 g of flowers (SFL2) or stigmas, respectively [14]. Regarding Cu, SFL1 had similar concentrations (0.57 ± 0.01 mg/100 g dw) compared to SFL2 (0.55 ± 0.04 mg/100 g dw), showing statistically no-significant differences between them. Except for Greek saffron (1.63 ± 0.11 mg/100 g dw), stigmas showed low amounts of Cu (0.25–0.35 mg/100 g dw). These differences could be also due to the cultivation conditions, but further research would be necessary. Therefore, saffron floral by-products and saffron stigmas showed high concentrations of macrominerals, representing an interesting nutritional quality parameter for the development of new food ingredients.

### Hydrophilic Compounds of Saffron and its Floral By-Products

Data on the organic acid composition of the saffron floral by-products and saffron stigmas of different origin are shown in Table 3. Organic acids play an important role in food since they are responsible for essential sensory properties but also have pharmacological actions [16]. Saffron stigmas presented the highest concentrations of lactic acid (5.27–6.02 g/100 g dw) with respect to the flowers, where it was significantly lower (3.59-4.0 g/100 g dw). However, saffron floral by-products had higher content of malic acid, especially SFL2 (5.89 ± 0.72 g/100 g dw), showing statistically significant differences with SFL1 and saffron stigmas, but saffron stigmas also presented elevated concentration of malic acid (2.03–2.72 g/100 g dw). Besides, saffron floral by-products contained propionic acid (2.13–2.34 g/100 g dw) and a low content of fumaric acid (0.17 g/100 g dw). Malic and fumaric acid were not detected in saffron stigmas. However, saffron stigmas showed the presence of formic acid, having the Greek saffron sample significantly higher amounts (7.18 ± 0.39 g/100 g dw) than Spanish and Iranian stigmas. Saffron samples had also oxalic acid, but in the lowest concentrations (around 0.30 g/100 g dw). Formic and oxalic acids were not detected in saffron floral by-products. Therefore, both saffron floral by-products and stigmas showed high amounts of lactic and malic acids, which could exert positive effects on human health and have other technological functionalities: lactic acid could act as preservative towards several spoilage and pathogenic microorganisms, and malic acid as acidulant [17]. Moreover, it should be noted that saffron flowers had propionic acid which has antimicrobial properties, being used as preservative in food [17]. These results are comparable to those obtained by Serrano-Díaz et al. [9] that showed the presence of lactic and malic acids in Spanish saffron bio-residues and the content of lactic and oxalic acid in saffron stigmas from Spain, and with those obtained by Pires et al. [11] that showed the content of malic acid in high amounts in edible petals from different species such as rose and calendula (1.23 ± 0.02 g/100 g dw and 1.14 ± 0.02 g/100 g dw, respectively). Besides, Jarukas et al. [18] observed the content of lactic, and malic acids in saffron spice from Iran.

**Table 3:** Organic acid and sugar composition in saffron and its floral by-products

	Samples				
	SFL1	SFL2	Spanish Saffron	Iranian Saffron	Greek Saffron
**Organic acids (g/100 g dw)**					
Lactic acid	3.59 ± 0.85b	4.00 ± 0.62bc	6.02 ± 0.16a	5.27 ± 0.43ac	5.55 ± 0.77a
Malic acid	3.86 ± 0.88b	5.89 ± 0.72a	2.72 ± 0.31b	2.57 ± 0.65b	2.03 ± 0.62bc
Fumaric acid	0.17 ± 0.03a	0.17 ± 0.01a	nd	nd	nd
Propionic acid	2.13 ± 0.85a	2.34 ± 0.60a	nd	nd	nd
Oxalic acid	nd	nd	0.32 ± 0.02a	0.28 ± 0.02a	0.29 ± 0.04a
Formic acid	nd	nd	4.42 ± 0.45b	4.65 ± 0.27b	7.18 ± 0.39a
**Soluble sugars (g/100 g dw)**					
Glucose	12.81 ± 1.23a	13.55 ± 1.49a	6.50 ± 0.06b	6.32 ± 0.14b	6.63 ± 0.28b
Fructose	28.67 ± 3.19b	35.33 ± 4.29a	2.82 ± 0.25c	1.58 ± 0.31c	0.63 ± 0.14c

Means ± standard deviation in the same line followed by different lowercase letters indicate statistically significant differences at (*p* ≤ 0.05) for each sample (n = 3); nd: not detected; SFL1, SFL2: Saffron floral by-products from two different producers

Regarding the composition of soluble sugars, all samples presented reducing sugars such as glucose and fructose monosaccharides (Table 3), which play an important role in food, not only in the sensory properties, but also in the food preservation. Besides, they represent a source of energy [19]. Saffron floral by-products had elevated glucose content (12.81–13.55 g/100 g dw) and high concentrations of fructose, especially SFL2 (35.33 ± 4.29 g/100 g dw), showing statistically significant differences with SFL1 and saffron stigmas. In saffron stigmas samples, the concentration of glucose was higher than fructose (6.32–6.63 and 0.63–2.82 g/100 g dw, respectively). These results are comparable to those of other edible plants, such as Dahlia and Rose petals, in which fructose was the soluble sugar found in the highest amount (⁓4–5 g/100 g dw), followed by glucose (3.23 g/100 g dw) [11].

### Lipophilic Compounds of Saffron and its Floral by-products

The fatty acid profile of saffron and its floral by-products is shown in Table 4. The study of fatty acid composition in food is very important since essential fatty acids, which must come from the diet, have an important functional role in human health [20].

Regarding saturated fatty acids, palmitic acid (C16:0) was found in the highest concentration in both saffron flower samples (20.19 g/100 g) and also in all saffron stigmas, with the highest proportion in Greek stigmas (15.18 ± 0.21 g/100 g). With respect to monounsaturated fatty acids, the quantitatively most important FAs were eicosanoic acid (C20:1n-9) and oleic acid (C18:1n-9) in saffron stigmas, as well as in the floral by-products. SFL1 presented higher proportion of eicosanoic acid (11.04 ± 0.38 g/100 g) and of oleic acid (2.57 ± 0.05 g/100 g), compared to SFL2 (5.56 ± 0.17 and 2.13 ± 0.25 g/100 g, respectively). Greek saffron stigmas showed the most elevated concentrations of eicosanoic acid (7.92 ± 0.26 g/100 g) and oleic acid (7.66 ± 0.17 g/100 g) respect to Spanish and Iranian samples. According to the recommendations of the European Food Safety Authority, the daily intake of SFA should be the lowest possible [21]. The proportion of polyunsaturated fatty acids were predominant in all samples, being linoleic acid (C18:2n6) the major FA found. Linoleic acid, an essential fatty acid produced in plants, is the most prevalent n-6 PUFA in the human diet. SFL2 presented an amount of linoleic acid (39.62 ± 2.83 g/100 g) higher than SFL1 (35.12 ± 0.26 g/100 g). Besides, Greek saffron stigmas (45.43 ± 1.08 g/100 g) showed an elevated concentration of linoleic acid, that was higher in Spanish (35.76 ± 0.72 g/100 g) and Iranian stigmas (40.24 ± 7.20 g/100 g). Traditional sources of linoleic acid are vegetable oils, such as sunflower oil containing around 65% of this FA whose consumption may reduce the risk of cardiovascular diseases. According to the recommendations of the European Food Safety Authority, the adequate intake per day of linoleic acid should be 4% of total energy intake [22].

**Table 4:** Fatty acid profile (main groups and ratios) for saffron and its floral by-products

	Samples				
Fatty acids (g/100 g total FAs)	SFL1	SFL2	Spanish Saffron	Iranian Saffron	Greek Saffron
Myristic acid (C14:0)	1.41 ± 0.03	3.63 ± 2.19	1.51 ± 0.03	2.45 ± 1.38	2.82 ± 1.13
Pentadecanoic acid (C15:0)	0.18 ± 0.00	0.37 ± 0.24	0.12 ± 0.00	0.22 ± 0.11	0.21 ± 0.02
Palmitic acid (C16:0)	20.19 ± 0.08	20.19 ± 2.09	12.40 ± 0.25	14.19 ± 2.62	15.18 ± 0.21
Stearic acid (C18:0)	2.56 ± 0.04	3.15 ± 0.85	1.57 ± 0.03	0.85 ± 0.17	0.89 ± 0.02
Behenic acid (C22:0)	1.64 ± 0.06	1.12 ± 0.03	0.49 ± 0.01	0.73 ± 0.13	0.93 ± 0.01
Lignoceric acid (C24:0)	2.26 ± 0.06	1.01 ± 0.02	0.18 ± 0.00	0.54 ± 0.12	0.56 ± 0.00
**∑ SFA**	**28.55 ± 0.19ab**	**29.64 ± 5.15a**	**16.27 ± 0.33c**	**18.98 ± 4.53bc**	**20.70 ± 1.03ac**
Hypogeic acid (C16:1n-9)	0.61 ± 0.00	1.27 ± 0.87	0.35 ± 0.01	0.85 ± 0.24	0.87 ± 0.16
Palmitoleic acid (C16:1n-7)	1.69 ± 0.01	2.02 ± 0.55	0.30 ± 0.01	0.40 ± 0.06	0.40 ± 0.05
Oleic acid (C18:1n-9)	2.57 ± 0.05	2.13 ± 0.25	4.39 ± 0.09	5.51 ± 0.94	7.66 ± 0.17
Vaccenic acid (C18:1n-7)	1.52 ± 0.02	1.35 ± 0.13	2.88 ± 0.06	2.93 ± 0.52	4.00 ± 0.08
Eicosanoic acid (C20:1n-9)	11.04 ± 0.38	5.56 ± 0.17	5.23 ± 0.10	6.37 ± 1.31	7.92 ± 0.26
Cis-14-tricosenoic acid (C23:1n-9)	5.59 ± 0.03	2.24 ± 0.45	0.38 ± 0.01	0.91 ± 0.18	0.96 ± 0.01
Erucic acid (C22:1n-9)	1.26 ± 0.02	0.55 ± 0.20	0.28 ± 0.00	0.35 ± 0.08	0.29 ± 0.00
Nervonic acid (C24:1n-9)	0.84 ± 0.02	0.63 ± 0.01	0.47 ± 0.01	0.77 ± 0.16	0.59 ± 0.01
**∑ MUFA**	**25.13 ± 0.37a**	**15.76 ± 2.22c**	**14.28 ± 0.29c**	**18.16 ± 3.39bc**	**22.79 ± 0.18ab**
Linoleic acid (C18:2n-6)	35.12 ± 0.26	39.62 ± 2.83	35.76 ± 0.72	40.24 ± 7.20	45.43 ± 1.08
γ-Linolenic acid (C18:3n-6)	nd	7.16 ± 0.82	25.95 ± 0.52	12.94 ± 1.30	nd
Eicosadienoic acid (C20:2n-6)	0.25 ± 0.03	0.10 ± 0.03	0.36 ± 0.01	0.19 ± 0.07	0.43 ± 0.00
Arachidonic acid (C20:4n-6)	nd	nd	0.26 ± 0.00	nd	0.35 ± 0.06
Docosadienoic acid (C22:2n-6)	0.15 ± 0.00	nd	0.93 ± 0.02	0.59 ± 0.03	1.12 ± 0.04
**∑ n-6 PUFA**	**35.94 ± 0.23b**	**47.12 ± 7.77ab**	**63.26 ± 1.27a**	**54.05 ± 10.13ab**	**47.33 ± 1.19ab**
α-Linolenic acid (C18:3n-3)	9.28 ± 0.40	6.87 ± 0.68	5.77 ± 0.12	7.76 ± 1.38	8.50 ± 0.10
Eicosatrienoic acid (C20:3n-3)	0.28 ± 0.04	0.33 ± 0.11	0.23 ± 0.01	0.50 ± 0.08	0.39 ± 0.03
**∑ n-3 PUFA**	**10.38 ± 0.41a**	**7.48 ± 0.39ab**	**6.19 ± 0.12b**	**8.80 ± 2.21ab**	**9.18 ± 0.35ab**
**∑ PUFA**	**46.31 ± 0.18b**	**54.60 ± 7.37ab**	**69.45 ± 1.39a**	**62.85 ± 7.92ab**	**56.52 ± 0.85ab**

Means ± standard deviation in the same line followed by different lowercase letters indicate statistically significant differences at (*p* ≤ 0.05) for each sample (n = 3); nd: not detected; SFL1, SFL2: Saffron floral by-products from two different producers

Regarding n-3 PUFA, the essential α-linolenic acid (C18:3n-3), mostly found in plant foods such as flaxseed, walnuts and vegetable oils, was predominant in saffron (5.77–8.50 g/100 g) and its floral-by-products (6.87–9.28 g/100 g). Thus, the diet richness in PUFA, replacing dietary SFA, contribute to lower LDL cholesterol and it is associated with a lower cardiovascular risk. These results were in accordance with other studies, showing the same tendency in fatty acids content of some edible plants, being polyunsaturated fatty acids group the predominant (46–57%) in petals from different species (dahlia, rose, calendula and centaurea) and were similar to the content of vegetable oils like soybean oil (6%) [11]. Therefore, due to the high content of n-6 and n-3 fatty acids and the lower content of SFA, the intake of saffron and its floral by-products may provide additional benefits for cardiovascular health.

### Expected Impacts of Saffron Waste Valorization

**Fig. 1:**
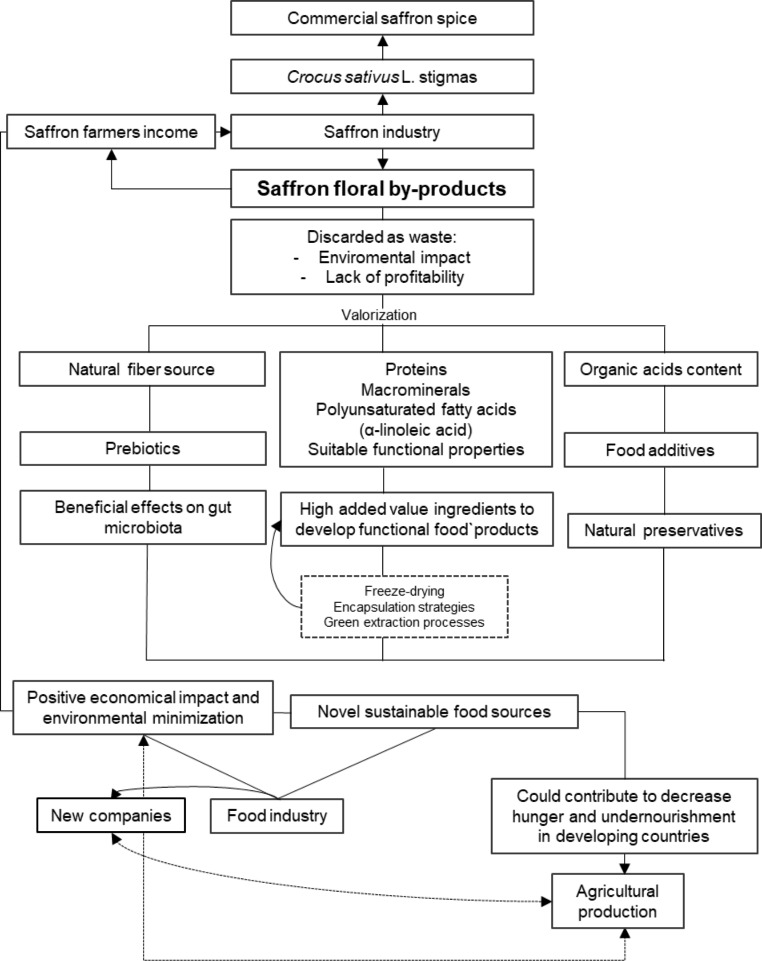
Diagram about the economic, social and environmental expected impacts from the valorization of saffron floral by-products

Currently, the management of food by-products through sustainable solutions is one of the main challenges of agro-food industries which are continuously searching for innovative solutions to obtain zero waste. As shown in Fig. 1, only the stigmas of *Crocus sativus* L. are used to obtain the commercial saffron spice. However, several tons of floral bio-residues are generated for each kg of spice, but these biomaterials are a source of valuable compounds such as proteins, PUFA, organic acids, and dietary fibers. Therefore, the valorization of these saffron floral by-products must take full advantage of their nutritional and functional potential in order to achieve economic, social and environmental positive impacts: (1) social benefits, since the use of this new food source as healthy and sustainable natural ingredients for human food may contribute to reduce hunger and undernourishment in developing countries, as well as contributing to the demand for new foodstuffs due to the growing world population; (2) financial benefits, since the added value generated in saffron floral by-products could create new companies, generating job opportunities and could be a new income for saffron farmers and for the saffron industry, which might lead to an increase in wages and the employment rate; (3) environmental impact minimization, since their valorization contributes to the reduction in waste accumulation, taking advantage of a biomass that is unexploited [7]. At the same time, saffron production will become more sustainable and profitable.

Therefore, saffron floral waste could be used as high-added value ingredients adding them into the food matrices previously freeze-dried to improve the safety of food by-products, separated individually from the biological matrix by green and sustainable strategies or by microencapsulation strategies to decrease any oxidation of bioactive, nutritional and functional components contained in them when in contact environmental factors [23]. In order to contribute to this approach, this study provides new information emphasizing the nutritional characterization of saffron floral by-products to unveil a deeper knowledge of their application as high-added value ingredients, which could generate economic gains for the industry, and contribute to reducing nutritional and environmental problems.

## Conclusion

This research provides new information on the nutritional value and composition of saffron spice and its floral by-products, in terms of hydrophilic and lipophilic compounds, revealing their potential as promising sources that could be further processed to be incorporated into different food matrices, due to their good composition of natural fiber, organic acids and soluble sugars, macrominerals and n-6 and n-3 fatty acids. In addition to the nutritional potential of saffron flowers, their suitable functional properties could increase the future perspectives to use them as sustainable ingredients. Nevertheless, further research would be necessary focused on the beneficial effects of saffron floral by-products on human health, such as intestinal health due to their fiber content or on cardiovascular health because of their content of n-6 and n-3 fatty acids. Furthermore, through the valorization of saffron floral by-products, this research could also contribute to the improvement of the sustainability of the saffron spice production and to the profitability of this agro-industrial sector taking advantage of a high-value biomass that is currently unexploited and discarded representing an environmental problem.

## Electronic Supplementary Material

Below is the link to the electronic supplementary material.

Supplementary Material 1

## Data Availability

Data available upon request.

## Abbreviations

DF: dietary fiber
dw: dry weight
FA: fatty acid
fw: fresh weight
MUFA: monounsaturated fatty acids
SFA: saturated fatty acids
SFL: saffron floral by-products
PUFA: polyunsaturated fatty acids
