# Supplementary material for: Underutilized Crocus Sativus L. Flowers: A Hidden Source of Sustainable High Value-Added Ingredients
Source: Plant Foods Hum Nutr. 2023 Jun 30;78(2):458–66. doi: 10.1007/s11130-023-01065-7 (PMC10363081; doi:10.1007/s11130-023-01065-7)
Supplement: Supplementary file 1 — Supplementary Material 1 [file 11130_2023_1065_MOESM1_ESM.docx]

**SUPPLEMENTARY MATERIAL**

**Material and Methods**

**Plant material**

Saffron floral by-products were obtained from two different producers (SFL1, SFL2) of a village belonging to the Toledo province (Castilla-La Mancha region, Spain), during the 2020 harvest season and cultivated following the requirements established by the Protected Designation of Origin “La Mancha Saffron” according to DOCM [1]. Saffron floral by-products were composed of all the parts of saffron flowers (tepals, stamens and styles), except for the stigmas that were detached manually from the rest of the flower by hand, following traditional procedures according to DOCM [1]. All fresh flowers were frozen in liquid nitrogen and kept at -80 °C until freeze-dried in a freeze-dryer Christ Alpha 2-4 (B. Braun Biotech International, Melsungen, Germany) for 48 h to constant weight. The conditions of the freeze dryer were: initial temperature -25 ± 2 °C and pressure 0.220 mbar. Then, they were crushed and sieved through a 500 μm mesh size, and stored in polyethylene bags at -20 °C until further analysis.

Stigmas were obtained from flowers of *Crocus sativus* L. from Spanish (Castilla-La Mancha region), Greek (Kozani area) and Iranian cultivations (Torbat zone), which were supplied by the Spanish company Verdú Cantó Saffron Spain, S.L.

**Proximal composition and dietary fiber**

The moisture and ash content of freeze-dried flower samples and saffron stigmas were determined according to ISO 3632 [2]. Protein content (Kjeldahl method using a conversion factor of 6.25), fat content and total dietary fiber, insoluble dietary fiber and soluble dietary fiber were estimated according to AOAC [3]. Available carbohydrates were calculated by the difference (meaning 100-the sum of moisture, ash, protein and fat), and the energy, total caloric values (kcal), were determined on the basis of a 100 g portion using values for protein (×4 kcal/g), carbohydrate (×4 kcal/g) and fat (×9 kcal/g) [4].

**Extraction organic acids and sugars**

The extractions were prepared using ultrapure water, and a sample/water ratio 1:20 (*w/v*) for the freeze-dried flowers and a ratio 1:50 (*w/v*) for saffron stigmas. The extracts were shaken for 1 h in the dark at 400 rpm on a magnetic stirrer at room temperature (Ovan, mod. MultiMix Heat D-MMH30E, Barcelona, Spain) and then sonicated for 15 min and centrifuged at 11200 x g for 10 min at 4 °C. Then, the supernatants were filtered (0.45 µm PTFE filter, Millipore, Spain) and stored at -20 °C. All extractions were done in triplicate.

**Analysis of organic acids and sugars**

The identification and quantification of sugars and organic acids were carried out by high performance liquid chromatography using Hewlett-Packard HPLC series 1100 equipment (Woldbronn, Germany) equipped with a Supelcogel C-610H column (30 cm x 7.8 mm) and a Supelcoguard C-610H pre-column (5 cm x 4.6 mm) (Supelco, Sigma Aldrich, St. Louis, MO, USA). The organic acids were measured at 210 nm in UV-VIS with diode array detector (DAD G1315A). For sugars, a refractive index detector (G1362A RID) was used. As a mobile phase, 0.1% orthophosphoric acid was used with an injection volume of 20 μl and the flow rate of 0.5 mL/min under isocratic conditions according to the method described by Cerdá-Bernad et al. [5]. The concentrations were calculated through calibration curves with the standards for sugars and organic acids (Sigma Aldrich, St. Louis, MO, USA). The results were expressed as mg/g of dry weight of sample.

**Minerals**

The minerals composition were determined according to Serrano-Díaz et al. [4], with slight modifications. Freeze-dried flower samples weighing 0.5 g were digested with 10 ml of 65 % HNO_3_ (*v/v*), and 0.1 g of saffron were digested with 5 ml of 65 % HNO_3_ (*v/v*) using a microwave reactor digestor (CEM Mars one, NC, USA) for 30 min with a temperature ramp whose final temperature was 200 °C. All samples were filtered (Whatman qualitative filter paper 90 mm) and diluted with ultrapure deionized water 1:50 (*v/v*) and stored at 4 °C. Total concentrations of macronutrients (Ca, Mg, Na and K) and micronutrients (Zn, Cu, Mn and Fe) in the previously mineralized samples were quantified with an Inductively Coupled Plasma Mass Spectrometer (ICPMS-2030, Shimadzu, Kyoto, Japan).

**Fatty acid profile**

Fatty acids were extracted from 0.5 g of freeze-dried flowers or saffron stigmas, by homogenizing them in a vortex with 20 ml of chloroform/methanol (2:1 *v/v*). Total lipids were extracted according to Folch et al. [6] and non-lipid impurities were removed by washing with 0.88% KCl (*w/v*). Fatty acid methyl esters (FAME) were prepared by acid-catalysed transesterification of total lipids according to the method described by Christie [7]. FAME were separated and quantified by gas–liquid chromatography using an SP™ 2560 flexible fused silica capillary column (100 m long, internal diameter of 0.25 mm and film thickness of 0.20 mm) (Supelco 2560 SPTM, Bellefone, PA, USA) in a Hewlett Packard 5890 gas chromatograph (Bellefone, PA, USA).

**Functional properties**

**Water-holding capacity and water solubility**

The water-holding capacity (WHC) was determined mixing 0.25 g of freeze-dried saffron floral by-products in 10 ml of distilled water during 1 min in a vortex mixer and keeping samples to hydrate at room temperature for 30 min, prior to centrifugation at 2000 x g for 30 min. Excess supernatant was decanted and WHC was expressed as g water/g dry sample.

Solubility was measured following the methodology reported by Garau et al. [8], as % loss in the initial freeze-dried sample weight, used previously to determine WHC, after the recovery of insoluble material (precipitate) after the centrifugation process.

**Oil-holding capacity**

The oil-holding capacity (OHC) was conducted according to Mallek-Ayadi et al. [9], mixing 0.25 g of freeze-dried saffron floral by-products in 10 ml of sunflower oil during 1 min in a vortex mixer, prior to centrifugation at 2000 x g for 30 min. Excess supernatant was decanted and OHC was expressed as g oil/g dry sample.

**Swelling capacity**

To study the swelling capacity (SC), 0.1 g of freeze-dried saffron floral by-products were added to 10 ml of distilled water and heated in a water bath at 60 °C for 30 min with stirring, following the methodology reported by Kusumayanti et al. [10]. Then, samples were centrifuged at 1000 x g for 15 min and the precipitate was weighed, calculating SC using the Eq. 1:

Swelling capacity (g/g) = precipitate weight/initial sample weight (1)

**Statistical analysis**

All determinations were done in triplicate. Results were expressed as the mean ± standard deviation. The mean comparisons were carried out using an analysis of variance (ANOVA) and by the Tukey multiple range test, using SPSS version 21.0 software package (SPSS Inc., Chicago, IL). The significant differences were established as (*p* ≤ 0.05).

**Results and Discussion**

**Functional properties of saffron floral by-products**

In order to use saffron floral by-products as new ingredients to develop enriched products, the study of their functional properties is very relevant to provide information regarding the interactions between components, structure and physicochemical properties of food components with the nature of the environment or food matrices [9]. The functional properties of freeze-dried saffron floral by-products are presented in Fig. S1.

WHC was evaluated in order to study the optimum amount of water to obtain a quality food product regarding its consistency. Samples showed statistically significant differences in WHC values, being around 15 g water/g dw for SFL1 and 10 g water/g dw for SFL2. These differences could be attributed to different concentration of hydrophilic compounds between SFL1 and SFL2. However, OHC values were similar in SFL1 and SFL2 (7 g oil/g dw), and statistically no-significant differences were found. It is essential to evaluate oil-holding capacity to use these ingredients in the formulation of foods with high content of fat and emulsion to improve their palatability and flavor retention (meat or bakery products) [11].

To develop bakery products, swelling capacity is a relevant quality parameter which could be influence by the particle size and the starch content of samples. Due to their high concentration in available carbohydrates, saffron floral by-products showed values around 12 g/g dw of SC, without finding statistically significant differences. These results were higher than that reported in other studies for different starch sources like potato flours, in which values ranged from 3.40 to 3.67 g/g [10].

In addition, water solubility is related to the digestibility of food in order to develop infant formula and food [11]. The results of solubility of saffron floral by-products indicated a high solubility which could be present a high digestibility, being excellent ingredients to develop new food products. Values ranged from 34 to 37 % for SFL2 and SFL1, respectively, showing statistically significant differences, which may be also due to the content of hydrophilic compounds.

Therefore, saffron floral by-products presented adequate and suitable functional properties, so they could be exploited as food ingredients to add in several food matrices developing novel functional food products with enhanced nutritional values and functional characteristics.


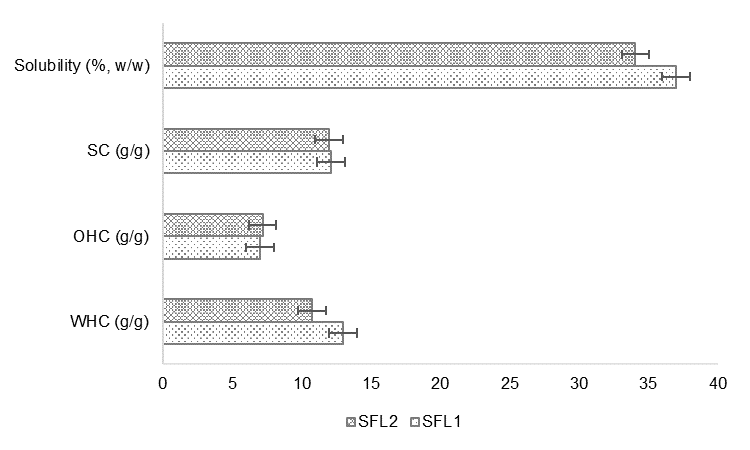


a

b

a

b

**Fig. S1** Functional properties of saffron floral by-products. Error bars represent standard deviation and different lowercase letters indicate statistically significant differences at (*p* ≤ 0.05) for each sample (n = 3); SFL1, SFL2: Saffron floral by-products from two different producers; SC: Swelling capacity; OHC: Oil-holding capacity; WHC: Water-holding capacity.

**References**

1. DOCM (1999) Specifications for the saffrons protected by this Denomination of Origin, which includes those saffrons produced in a defined geographical area of the Autonomous Community of Castilla-La Mancha. Official Gazette of Castilla-La Mancha 19:1098-112.

2. ISO 3632 (2011) Part 1: Specification, Part 2: Test Methods, International Organization for Standardization, Geneva

3. AOAC (1995) Official Methods of Analysis. 16th ed. Association of Official Analytical Chemists, Washington, DC.

4. Serrano-Díaz J, Sánchez AM, Martínez-Tomé M, Winterhalter P, Alonso GL (2013) A contribution to nutritional studies on *Crocus sativus* flowers and their value as food. J Food Compost Anal 31:101-8. <https://doi.org/10.1016/j.jfca.2013.03.009>

5. Cerdá-Bernad D, Valero-Cases E, Pastor JJ, Frutos MJ, Perez-Llamas F (2021) Probiotic red quinoa drinks for celiacs and lactose intolerant people: study of functional, physicochemical and probiotic properties during fermentation and gastrointestinal digestion. Int J Food Sci Nutr 1-11. <https://doi.org/10.1080/09637486.2021.1921707>

6. Folch J, Lees M, Sloane Stanley GH (1957) A simple method for the isolation and purification of total lipides from animal tissues. J Biol Chem 226:497-509.

7. Christie WW (2003) Lipid analysis: isolation, separation, identification and structural analysis of lipids, The Oily Press, Bridgewater

8. Garau MC, Simal S, Rosselló C, Femenia A (2007) Effect of air-drying temperature on physico-chemical properties of dietary fibre and antioxidant capacity of orange (*Citrus aurantium* v. Canoneta) by-products. Food Chem 104:1014-24. <https://doi.org/10.1016/j.foodchem.2007.01.009>

9. Mallek-Ayadi S, Bahloul N, Kechaou N (2017) Characterization, phenolic compounds and functional properties of *Cucumis melo* L. peels. Food Chem 221:1691-7. <https://doi.org/10.1016/j.foodchem.2016.10.117>

10. Kusumayanti H, Handayani NA, Santosa H (2015) Swelling power and water solubility of cassava and sweet potatoes flour. Procedia Environ Sci 23:164-7. <https://doi.org/10.1016/j.proenv.2015.01.025>

11. Awuchi CG, Ogwe VS, Echeta CK (2019) The functional properties of foods and flours. Int J Adv Acad Res 5:139-60.
